# Structural properties and enzyme stabilization function of the intrinsically disordered LEA_4 protein TdLEA3 from wheat

**DOI:** 10.1038/s41598-019-39823-w

**Published:** 2019-03-06

**Authors:** Sana Koubaa, Anne Bremer, Dirk K. Hincha, Faiçal Brini

**Affiliations:** 1 Biotechnology and Plant Improvement Laboratory, Centre of Biotechnology of Sfax (CBS)/University of Sfax, B.P ‘’1177”, 3018 Sfax, Tunisia; 2 Max-Planck-Institute of Molecular Plant Physiology, Am Mühlenberg 1, D-14476 Potsdam, Germany; 3 Present Address: St. Jude Children’s Research Hospital, 262 Danny Thomas Place, Memphis, TN USA

## Abstract

Late Embryogenesis Abundant (LEA) proteins are mostly predicted to be intrinsically disordered proteins (IDPs) that are induced under conditions of cellular dehydration. Their functions, however, are largely unexplored and also their structure and interactions with potential target molecules have only recently been investigated in a small number of proteins. Here, we have characterized the wheat LEA protein TdLEA3, which has sequence homology with the group of LEA_4 proteins that are characterized by the 11-mer repeat motif TAQAAKEKAXE. TdLEA3 has five repeats of this imperfectly conserved 11-mer amino acid motif. To investigate the structure of the protein, we used circular dichroism (CD) and Fourier-transform infrared (FTIR) spectroscopy. The data show that TdLEA3 was largely disordered under fully hydrated conditions and acquired α-helical structure upon drying and in the presence of trifluoroethanol (TFE). Moreover, the addition of increasing glycerol concentrations to the protein solution induced a progressive gain in α-helix content. Activity assays indicated that TdLEA3 was able to prevent the inactivation of lactate dehydrogenase (LDH) under heat, dehydration-rehydration and freeze-thaw treatments. In addition, TdLEA3 reduced aggregate formation in the enzyme during these treatments.

## Introduction

During their maturation phase, orthodox seeds lose most of their water content as part of their normal development. This maturation drying induces the accumulation of Late Embryogenesis Abundant (LEA) proteins. LEA proteins were first described more than 35 years ago, when they were found to be highly abundant during the late stages of cotton seed development, when the embryo becomes desiccation tolerant^1^. Subsequently, related proteins were found not only in the seeds of all other investigated plant species, but also in other plant tissues, in some bacterial species and in invertebrate animals such as nematodes, rotifers and brine shrimp^2^. According to their amino acid sequences, LEA proteins are classified into eight Pfam families based on conserved motifs^3,4^. The LEA_4 (PF02987) proteins, also referred to as Group 3 proteins^5^, are accumulated by plants, microorganisms and invertebrates in response to dehydration stress^6–9^.

LEA_4 proteins are hydrophilic and have been predicted to belong to the group of intrinsically disordered proteins (IDPs)^7,8^. This prediction has been confirmed for all LEA_4 proteins investigated experimentally^8,10^. However, their flexibility allows them to adopt mainly α-helical structure upon drying^8,10,11^. Recent studies on LEA proteins have focused on their structural transitions upon dehydration and the relationship of these transitions to possible functions. Structural analyses by circular dichroism (CD), nuclear magnetic resonance (NMR) and Fourier-transform infrared (FTIR) spectroscopy methods showed that several LEA proteins fold into α-helices in the presence of the chemical helix inducer trifluoroethanol (TFE) and when subjected to molecular crowding in the presence of glycerol^12–15^ or partial dehydration at low relative humidity^16^. Moreover, the addition of sodium dodecyl sulfate (SDS) induced α-helicity in several LEA proteins^7,8^ and NMR analyses indicated direct interactions of a dehydrin with this membrane-mimicking detergent^17^. Also, LEA18 from *Arabidopsis thaliana* showed partial folding into β-sheet conformation in the presence of liposomes^18^, while LEA7 showed partial folding into α-helices^19^. COR15A and two other LEA_4 proteins, on the other hand, only showed increased α-helicity in the presence of high concentrations of glycerol, indicating that partial folding was necessary for membrane interaction^15^.

LEA_4 proteins are able to function as protectants for enzymes during freezing or drying in addition to protecting membranes^20–27^. The ability of LEA proteins to protect the activity of desiccation sensitive enzymes from the deleterious effects of dehydration can, at least partially, be attributed to an ability to prevent enzyme aggregation^21,28,29^. It has been proposed that LEA proteins may act as a molecular shield to prevent protein aggregation. Their disordered and flexible structure could allow them to function as a physical barrier between enzyme molecules and thus prevent their aggregation under conditions that induce (partial) unfolding. In agreement with this proposed mechanism, it has been shown that several LEA proteins are able to reduce the aggregation of polyglutamine (polyQ) or amyloid ß-peptides when co-expressed in mammalian cells^30–32^.

Here, we have investigated the structural and functional properties of the LEA_4 protein TdLEA3 from durum wheat (*Triticum turgidum* L. subsp. *durum*). This protein shows 83% and 98% amino acid sequence identity to TaLEA2 and TaLEA3, respectively, from bread wheat (*Triticum aestivum* L.), which have, however, not been structurally and functionally characterized^33^. Here, we show that TdLEA3 is an IDP in dilute aqueous solution and folds into α-helices during drying and under conditions of glycerol-induced crowding. TdLEA3 was able to prevent the inactivation and aggregation of the enzyme lactate dehydrogenase (LDH) under heat, dehydration or freeze-thaw treatments indicating its potential function in cellular stress tolerance. To the best of our knowledge, TdLEA3 is the first LEA_4 protein for which chaperone activity, i.e. stabilization of an enzyme during heat stress in solution, has been shown.

## Results

### Production and Purification of Recombinant TdLEA3

**Figure 1:**
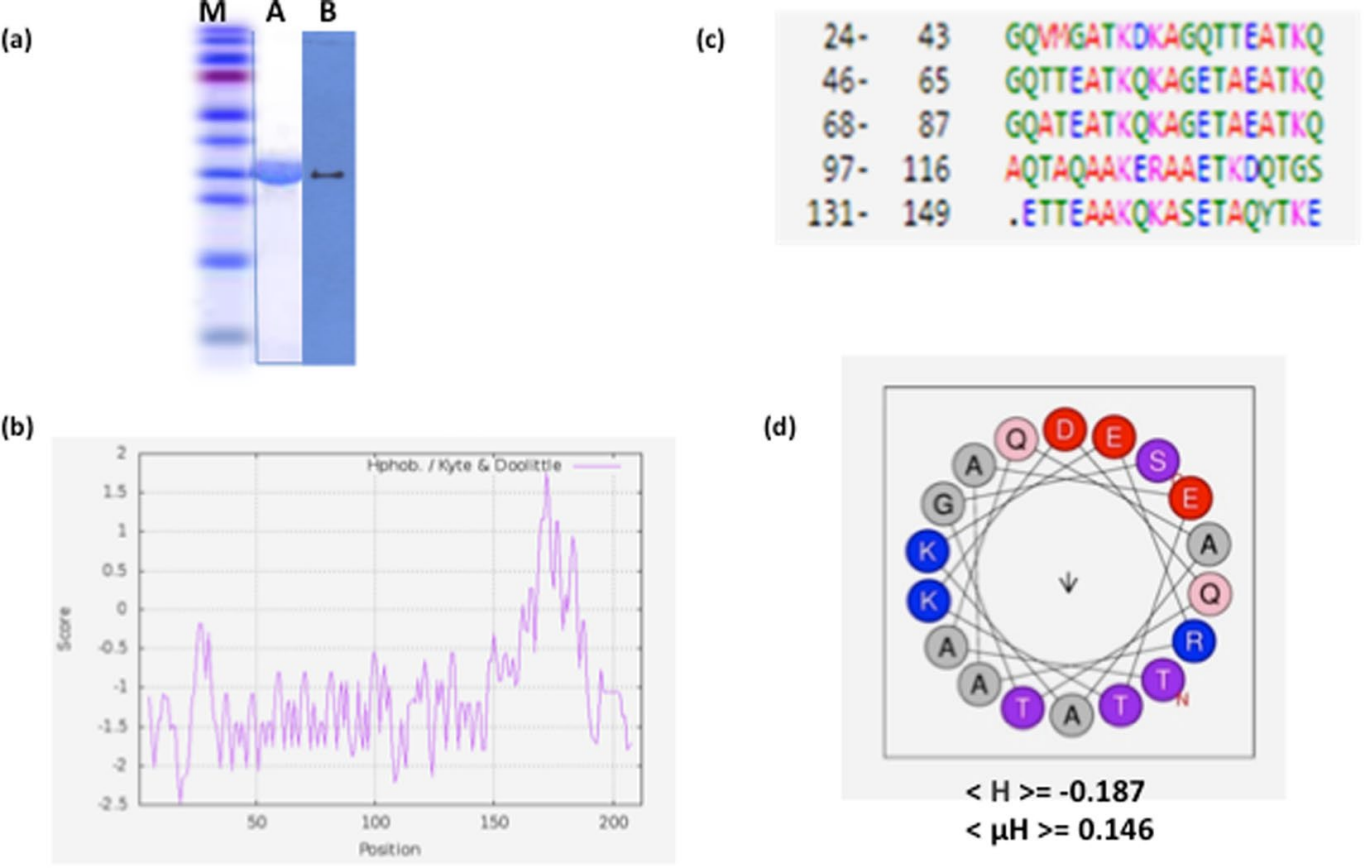
Purity and properties of the TdLEA3 protein. (**a**) Molecular mass and purity of TdLEA3. M. Size markers. A. SDS-PAGE analysis of recombinant TdLEA3. B. Western blot of TdLEA3 protein identified using a His_6_-tag specific antibody. (**b**) Hydropathy analysis of TdLEA3 using the Kyte-Doolittle scale. Regions with values above 0 are hydrophobic in character. (**c**) Alignment of the repeating 11-mer motifs found in TdLEA3 protein sequence using the RADAR software. Numbers refer to the amino acid position of the first residue in the motif. Green indicates polar amino acids, red hydrophobic residues, blue negatively charged residues and pink positively charged residues. (**d**) Helical wheel projection of the 11-mer repeat 68–87 region of TdLEA3. Red indicates negatively charged, blue positively charged, grey hydrophilic amino acids, (µH) is the mean hydrophobic moment and (H) the mean hydrophobicity of the sequence domain.

The open reading frame of *TdLEA3* was cloned in frame with the polyhistidine tag of the pET28a expression vector. Recombinant TdLEA3 protein was expressed in *E*. *coli* cells and purified by affinity chromatography. The purity of the protein was assessed by SDS-PAGE and western blotting (Fig. 1a). The protein migrated at a position in the gel corresponding to a higher molecular mass (35 kDa) than calculated from the amino acid sequence (21.9 kDa; Table 1), which has been frequently observed for LEA proteins^34^. The identity of TdLEA3 was verified by western blot analysis using an anti-His_6_ antibody (Fig. 1a).

**Table 1:** Physico-chemical properties of TdLEA3.

Number of amino acids	Molecular mass (kDa)	Theoretical pI	Grand average of hydropathy (GRAVY)	Predicted α‐helical content [%] (SOPMA)	Tendency of disorder (%) (IUPRED)
212	21.9	9.02	−1.096	81	76

### TdLEA3 amino acid composition and properties

*In silico* analysis of TdLEA3 using IUPRED indicated a high predicted level of disorder (76%), while the SOPMA tool predicted an 81% content of α-helices (Table 1). The amino acid composition of TdLEA3 is dominated by hydrophilic and charged amino acids. Consequently, the protein is highly hydrophilic, with a GRAVY of −1.096 and has a pI of 9.02. Also, a hydropathy analysis (Fig. 1b) based on the Kyte–Doolittle scale^35^ showed that the N-terminal 168 amino acids of the protein and the C-terminus (amino acids 185–208) are hydrophilic. However, there is an intervening hydrophobic domain that is rather atypical of LEA_4 proteins. Typical LEA_4 proteins contain one or several 11-mer repeat sequences TAQAAKEKAXE. TdLEA3 has five repeats of an imperfect 11-mer amino acid motif that are not identical to the consensus sequence (Fig. 1c). HELIQUEST predicts that the 11-mer repeat located in the region between amino acids 68 and 87 forms an amphipathic α-helix (Fig. 1d). Such helices have previously been suggested to be involved in interactions of LEA_4 proteins with target structures such as membranes^15^.

### TdLEA3 has an intrinsic propensity to fold into an α-helical conformation

**Figure 2:**
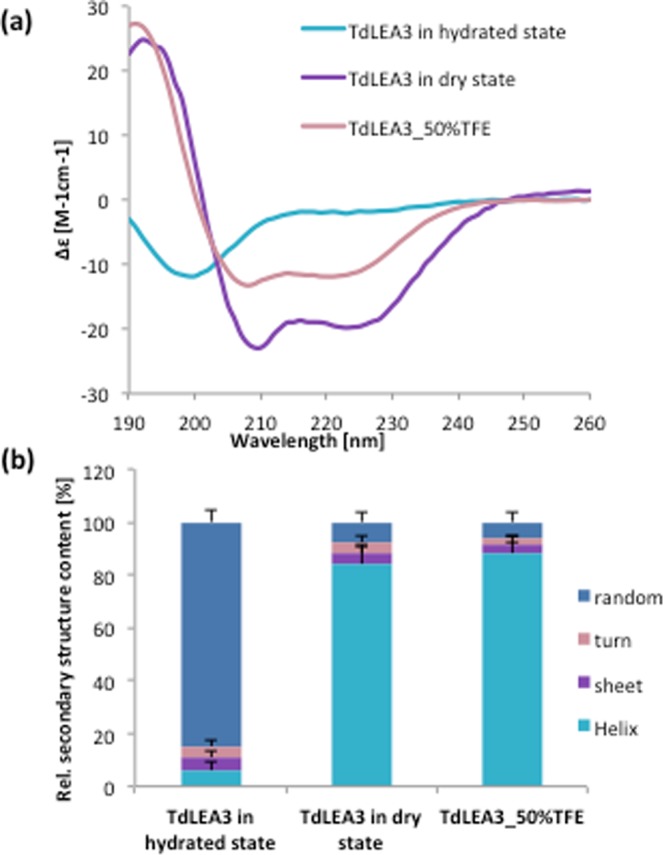
Far UV-CD analysis of the secondary structure of TdLEA3. (**a**) CD spectra of the protein in the hydrated state, in the dry state and in the presence of 50% of trifluoroethanol (TFE), (**b**) Secondary structure composition of TdLEA3, as calculated from the respective CD data for the hydrated protein, the dried protein and the protein exposed to 50% TFE. Error bars represent ± SEM from three different samples.

Given the diverging structural predictions obtained from IUPRED and SOPMA (Table 1), the structural features of TdLEA3 were further investigated by CD spectroscopy. The far UV-CD spectrum of TdLEA3 under fully hydrated conditions showed a negative ellipticity around 200 nm, indicating a largely unstructured conformation, typical for IDPs (Fig. 2a). However, after drying, the spectrum changed drastically and showed two minima at 208 and 222 nm, typical of a mainly α-helical conformation. A similar spectrum was also obtained in the presence a chemical inducer of α-helicity, trifluoroethanol (TFE). Secondary structure estimates obtained from these spectra indicated that TdLEA3 was approximately 84% random coil in the fully hydrated state, while both drying and the presence of 50% TFE induced over 80% α-helix content in the protein (Fig. 2b).

**Figure 3:**
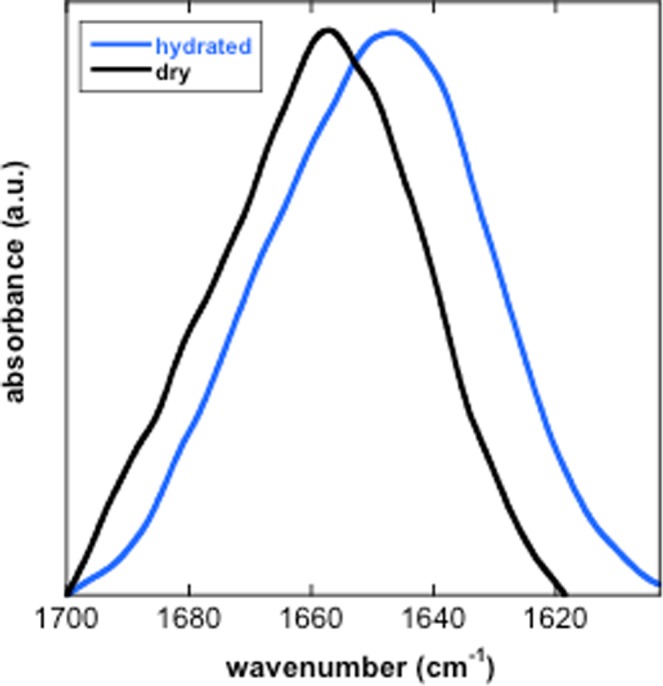
FTIR analysis of the secondary structure of TdLEA3. The protein was investigated in the hydrated (D_2_O) and in the dry state and the Amide I region of the FTIR spectra is shown as a mean of three samples. The Amide I peak of the fully hydrated protein was located at 1648 cm^−1^ indicating a highly disordered protein, while in the dry state the peak was shifted to 1657 cm^−1^ indicating largely α-helical conformation.

FTIR spectroscopy was used to further probe the secondary structure of TdLEA3. H_2_O gives rise to a strong absorbance peak in the FTIR spectrum around 1645 cm^−1^ that overlaps with the Amide I peak, which is indicative of protein secondary structure. Therefore, the protein was dissolved in D_2_O to avoid this interference. The broad Amide I peak (between approximately 1700 and 1600 cm^−1^) is composed of several component peaks that originate from different secondary structures. Bands in the region between 1660–1650 cm^−1^ are assigned to α-helix, while a peak in the region between 1640 cm^−1^ and 1650 cm^−1^ indicates unordered regions in a protein. A peak at around 1620 cm^−1^ is associated with intermolecular β-sheet aggregates^36–38^. The Amide I peak of the hydrated protein was centered at 1648 cm^−1^, indicating a mainly unstructured protein. Upon drying, this maximum was shifted to 1657 cm^−1^ (Fig. 3), indicating a gain in α-helix conformation during drying, in agreement with the CD spectra. We did not observe any indication for β-sheet aggregates.

### Low water availability due to glycerol-induced crowding or dehydration leads to folding of TdLEA3

**Figure 4:**
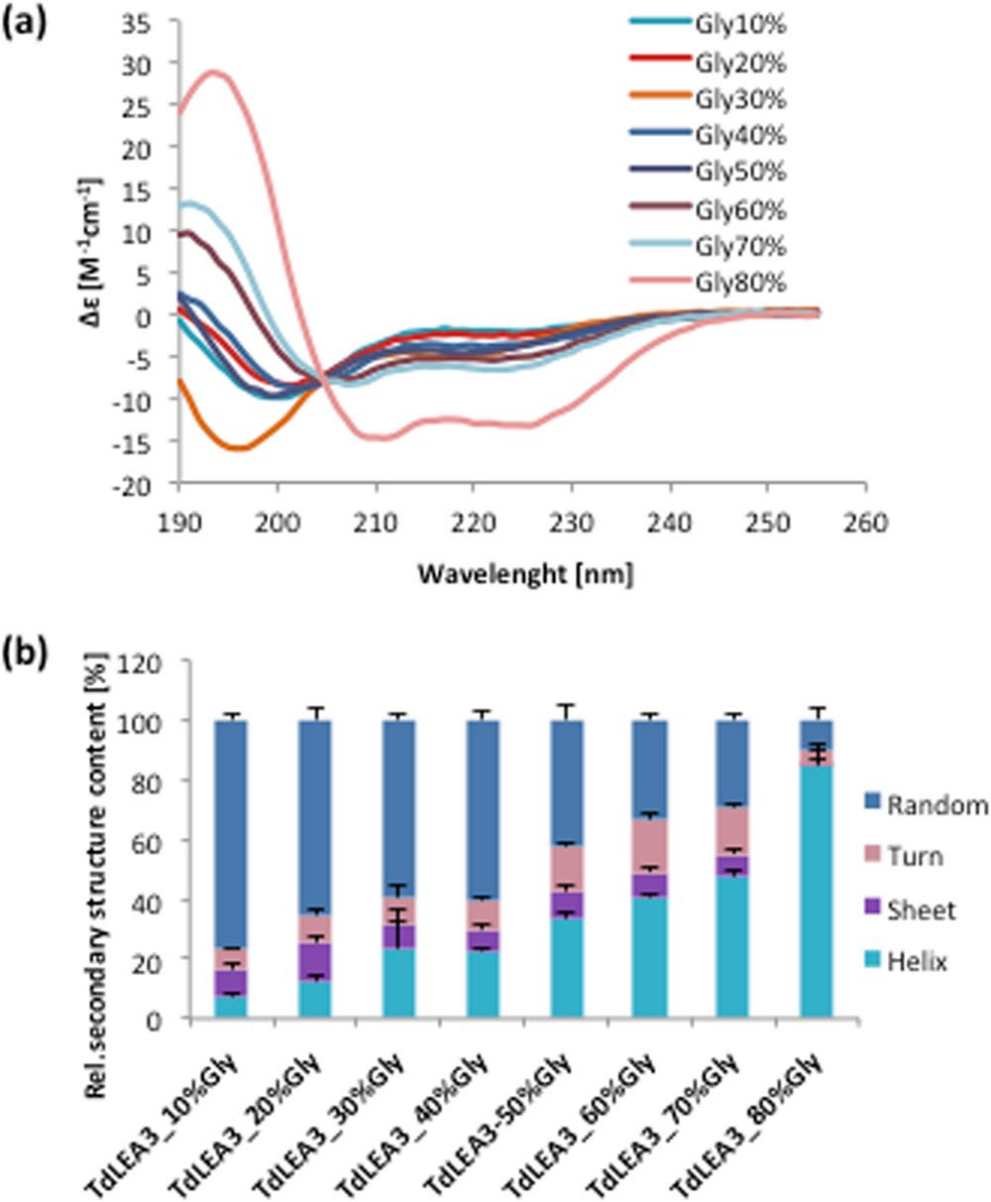
Effect of glycerol-induced crowding on the secondary structure of TdLEA3. (**a**) Far-UV CD spectra and (**b**) relative secondary structure content of TdLEA3 in the presence of increasing glycerol concentrations (10–80%; v/v). Error bars represent ± SEM from three samples.

It has been shown previously that some intrinsically disordered LEA proteins fold into α-helices in the presence of high concentrations of glycerol^12,13,15^. CD spectroscopy showed that TdLEA3 also folded in the presence of glycerol, from about 7% α-helix in 10% glycerol to 85% α-helix in 80% glycerol (Fig. 4).

**Figure 5:**
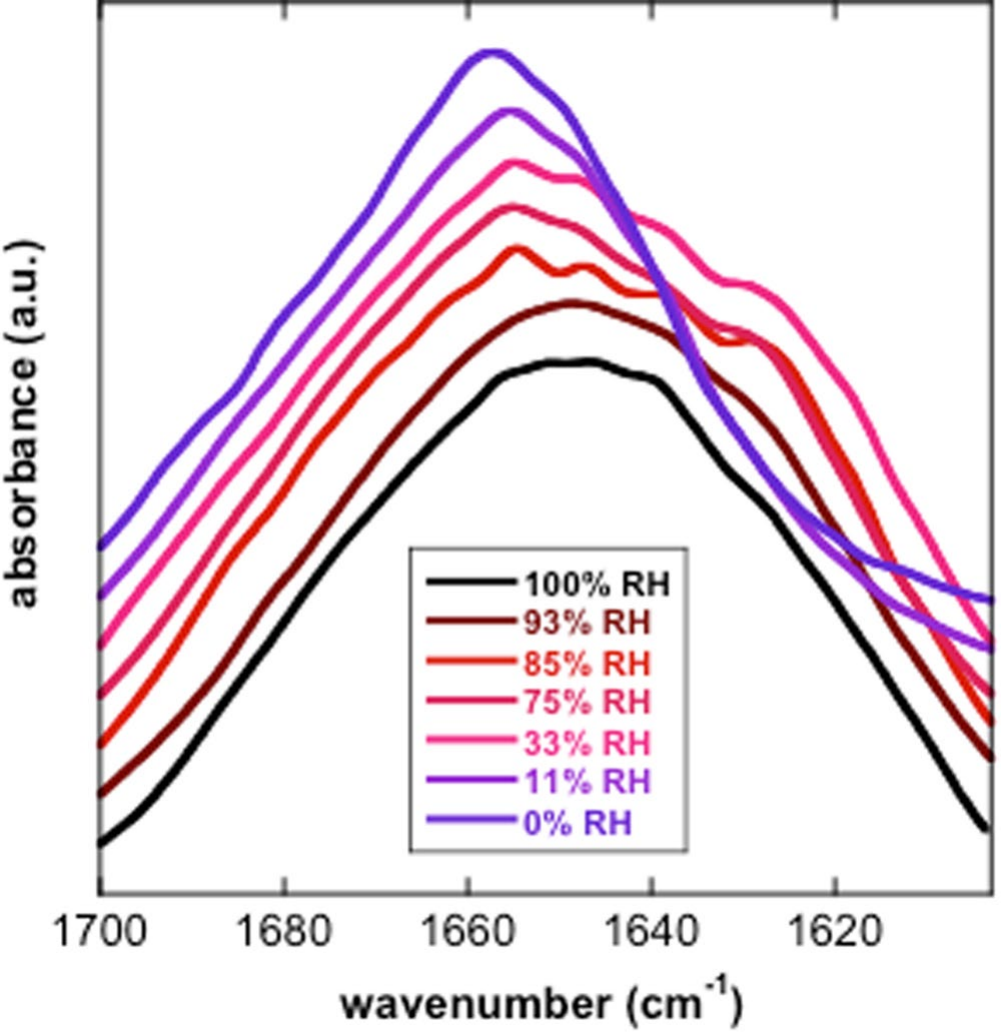
FTIR analysis of the secondary structure of TdLEA3 at different relative humidities (RH). Dry protein samples were equilibrated over different saturated salt solutions or over D_2_O (100% RH) producing the indicated RH. The Amide I peaks of TdLEA3 at different RH are shown as means of three samples each and are vertically off-set to improve the visibility of the curves.

We further explored the protein conformational transition by rehydrating the dry protein under conditions of different relative humidity. The secondary structure of TdLEA3 was recorded by FTIR spectroscopy under increasing humidity as previously reported^16^. In the dry state, the protein was characterized by a peak centered at about 1657 cm^−1^, indicating a largely α-helical protein, as indicated in Fig. 3. Equilibration of the protein at 11% RH only induced a minor shift in the Amide I peak. With further increases in RH, the Amide I peak broadened on the low wavenumber side, indicating partial unfolding. In addition, a shoulder at about 1624 cm^−1^ suggested partial aggregation under these conditions. At the two highest RH (93% and 100%) aggregation was no longer visible and the spectra were centered at about 1647 cm^−1^, in agreement with a disordered protein (Fig. 5).

### TdLEA3 stabilizes the enzyme LDH under different stress conditions

**Figure 6:**
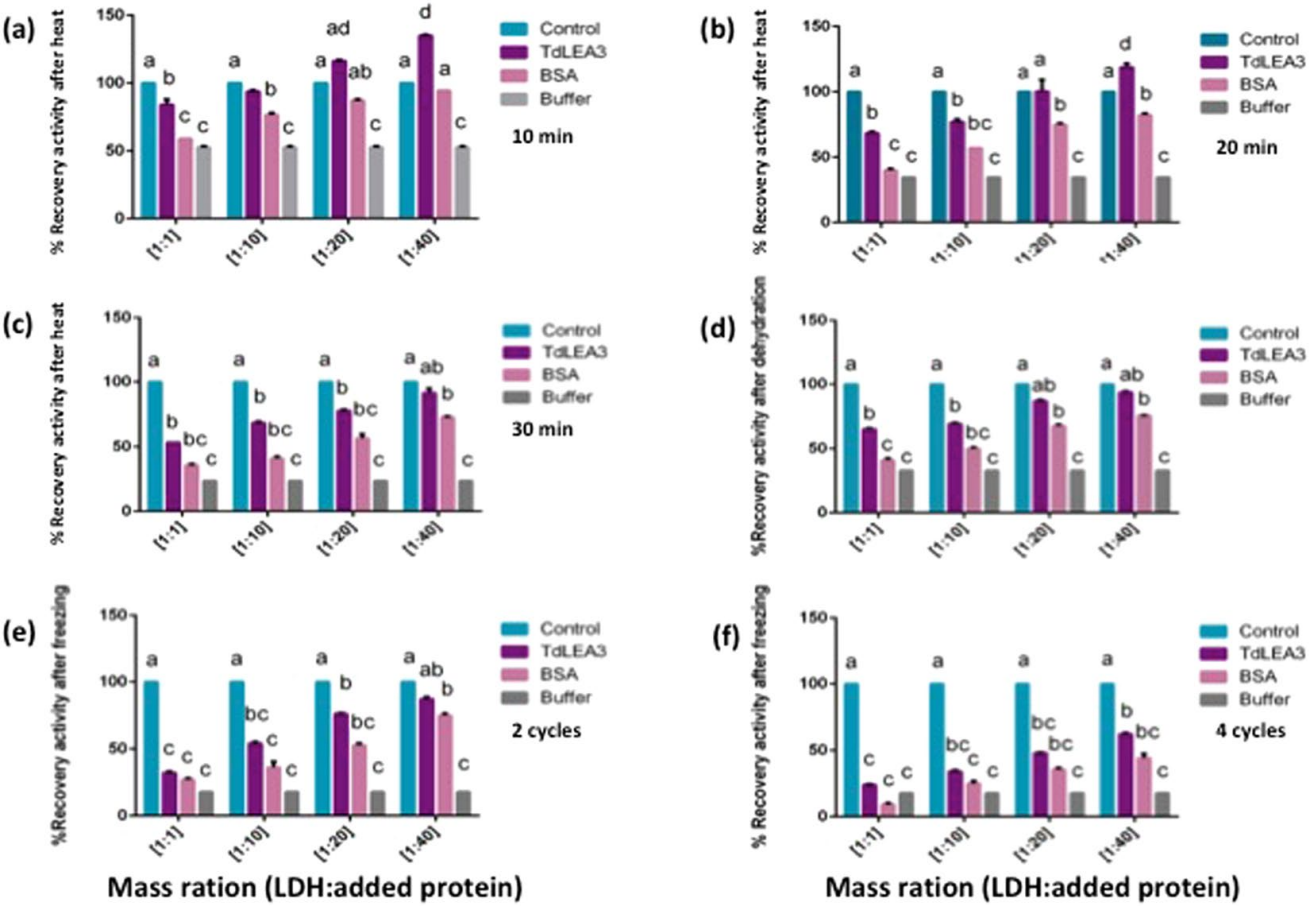
TdLEA3 protects lactate dehydrogenase (LDH) against inactivation under stress. (**a**–**c**) recovery of LDH activity during heating at 48 °C for the indicated times in the presence of TdLEA3, BSA, or buffer at the indicated mass ratios. (**d**) The recovery of LDH activity after dehydration in the presence of TdLEA3, BSA, or buffer at the indicated mass ratios. (**e**,**f**) Recovery of LDH activity following 2–4 freeze–thaw cycles in the presence of TdLEA3, BSA, or buffer at the indicated mass ratios. Throughout the experiments, addition of buffer was used as a negative control to assess the dilution effect. Data are expressed as % activity measured before the stress in the presence of the corresponding protein and are the averages ± SEM of three to five replicates from two independent experiments. Different letters indicate a significant difference (p-value < 0.05) evaluated with the Student’s t test.

LEA proteins confer *in vitro* stabilization of several enzymes under stress conditions (see^2,4^ for reviews). Therefore, we tested the ability of TdLEA3 to prevent the loss of LDH activity after heating, dehydration and freezing. We compared the effects of TdLEA3 with the effects of BSA as an example of a non-specific protectant and with LDH treated in buffer without additional protein. Under all stress conditions and at all concentrations, TdLEA3 provided a higher degree of protection for LDH than BSA, indicating that the LEA protein had a protective activity that went beyond the unspecific effects of having a second protein present (Fig. 6). After heating at 48 °C for 10 min, LDH had lost about half of its activity in buffer and activity was further reduced with longer incubation times, to about 20% after 30 min. In contrast, the enzyme activity was completely preserved after 10 or 20 min of heat treatment in the presence of TdLEA3 at mass ratios (LDH: TdLEA3) of 1:20 and 1:40. At the highest mass ratio TdLEA3 preserved more than 90% of the enzyme activity after 30 min at 48 °C.

After dehydration and rehydration, LDH activity was reduced to about 35% of its initial value. TdLEA3 provided strong stabilization to the enzyme at all mass ratios, reaching 93% of the fresh activity at a mass ratio (LDH: TdLEA3) of 1:40 (Fig. 6d). Two cycles of freezing and thawing were sufficient to reduce LDH activity massively and after four cycles, residual activity was below 20%. In the presence of the TdLEA3, LDH activity was partially protected. While at a mass ratio of 1:40 residual activity was close to 90% after two freeze-thaw cycles, it still reached 62% after four cycles (Fig. 6e,f).

### TdLEA3 inhibits LDH aggregation during stress treatments

**Figure 7:**
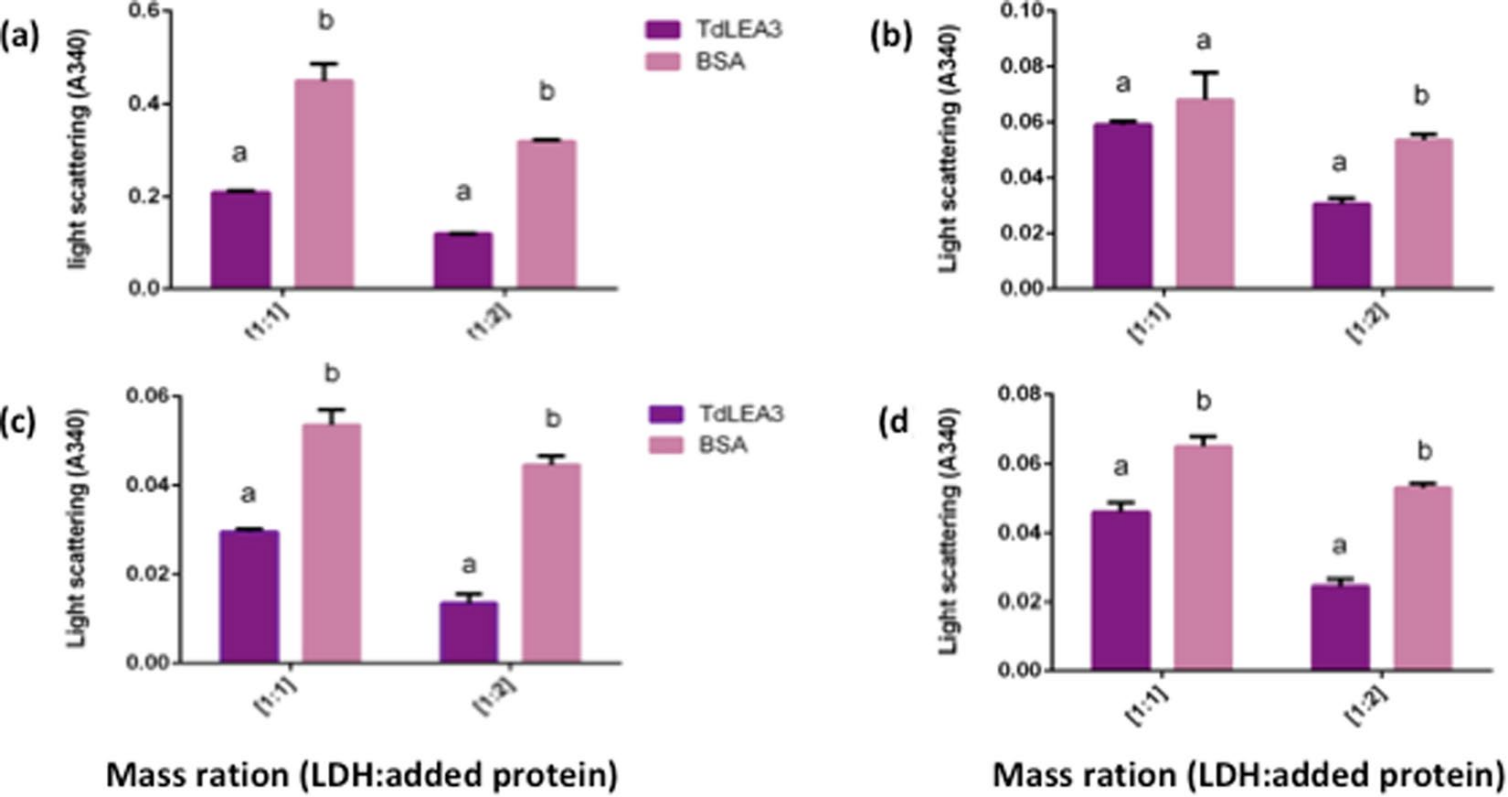
Anti-aggregation activity of TdLEA3. Heating (**a**), dehydration-rehydration (**b**), and two (**c**) or three freeze-thaw cycles (**d**) were used. Aggregation was measured in a spectrophotometer as the absorbance at 340 nm. TdLEA3 and BSA were added to the LDH at the indicated mass ratios. The data are the averages ± SEM from three independent experiments. Different letters indicate a significant difference (p-value < 0.05) evaluated with the Student’s t test.

LDH is well known to form aggregates when subjected to dehydration, heating or freeze-thaw treatments (see^39^ for a review). We therefore investigated the ability of TdLEA3 to reduce LDH aggregation during stress treatments by measuring apparent light scattering of protein solutions as absorbance. We used two mass ratios of enzyme: protein (1:1 and 1:2). After heating for 20 min at 80 °C, LDH showed massive aggregation. The presence of TdLEA3 reduced the aggregation of LDH at both investigated mass ratios to a larger extent than BSA (Fig. 7a). In the dehydration-rehydration assay, the absorbance in the presence of TdLEA3 and BSA was very similar at a mass ratio 1:1, but at a mass ratio 1:2 TdLEA3 reduced aggregate formation more strongly than BSA (Fig. 7b). During freezing and thawing, both BSA and TdLEA3 provided strong protection for LDH after both two and three cycles of freezing-thawing treatment at both mass ratios (Fig. 7c,d). However, LDH aggregation was consistently lower in the presence of the LEA protein than in the presence of the non-specific protectant BSA.

## Discussion

Both prediction (e.g.^40^) and spectroscopic investigations of recombinant proteins^7,8,10^ have indicated that LEA_4 proteins from different organisms lack stable secondary structure in dilute solution. This lack of structure places LEA proteins within a large class of proteins most commonly called intrinsically disordered proteins, or IDPs^41^. Among the most intensively studied plant IDPs are LEA proteins^42^. In an effort to provide additional insights into the structure and function of LEA_4 family proteins, we report the characterization of the wheat LEA protein TdLEA3. In agreement with its likely nature as an IDP, the protein is largely hydrophilic, with the exception of a small stretch of amino acids close to the C-terminus. To reveal the functional relevance of this unusual feature in a LEA_4 protein, mutational studies will be necessary. Also, the functional role of the amphipathic α-helix in TdLEA3 will require further research.

Bioinformatic prediction of TdLEA3 secondary structure resulted in widely diverging results in that IUPRED predicted the protein to be 76% disordered, while SOPMA predicted 81% α-helix content. Our CD and FTIR data clearly showed that recombinant TdLEA3 was predominantly disordered in dilute solution, but folded into α-helices during dehydration or under crowded conditions induced by high concentrations of glycerol. In addition, TdLEA3 gained α-helical structure in the presence of 50% TFE. This chemical agent induces protein folding by desolvating the protein backbone^43^, similar to drying or high concentrations of glycerol^14^. This also explains the prediction of a high degree of helicity in TdLEA3 by SOPMA, as this tool predicts structure in vacuo and therefore tends to predict the structure of dry LEA proteins rather than the hydrated disorder^15,19,44,45^.

Physiologically, it may be argued that LEA proteins such as TdLEA3 that are involved in the freezing or dehydration tolerance of plants should be inactive under non-stress, fully hydrated conditions, where they are unstructured, but may become activated through folding when water is lost from the cells due to evaporation or extracellular ice crystal formation. Increasing solute concentrations during dehydration were simulated *in vitro* with glycerol. Concentration-dependent folding in the presence of glycerol has also been shown for intrinsically disordered LEA proteins from other families^13,46^ and other osmolytes such as TMAO, sucrose and proline may have similar folding effects^47,48^. In line with this argument, our FTIR spectroscopy data showed a clear influence of relative air humidity on TdLEA3 folding. Such an effect has been shown previously for the LEA_4 protein COR15A from *Arabidopsis thaliana*^16^ which showed significant α-helicity at 97% RH. TdLEA3, on the other hand, was completely unfolded at 93% RH and only showed secondary structure at 85% RH or lower, indicating that different LEA_4 proteins differ in their propensity for dehydration-induced folding. This has not been observed previously as TdLEA3 is only the second LEA protein that has been investigated in this way and may open an interesting area of future research.

We have shown that TdLEA3 is able to protect the stress-sensitive target enzyme LDH from damage during drying, freezing and heat treatment. The protection afforded by the LEA protein was better than that provided by BSA, which is a well-known unspecific enzyme stabilizer. TdLEA3 did not only protect LDH from loss of catalytic activity, but also prevented enzyme aggregation. Such a reduction of enzyme aggregation, in particular during drying, has been observed for other LEA_4 proteins before and has been explained on the basis of the “molecular shield” hypothesis^21,27,28,49^. It should be stressed, however, that aggregation of LDH during drying depends strongly on the experimental conditions and that LDH can be completely inactivated by drying in the absence of any aggregation^50^. LDH activity was nevertheless protected under these conditions by the LEA_4 protein LEA7, indicating that protection of enzymes by LEA_4 proteins may be due to more than one mechanism.

Quite strikingly, TdLEA3 protected LDH not only during drying and freezing, activities that have been reported frequently for LEA proteins in the literature, but also under heat stress. To the best of our knowledge, such a chaperone activity has previously only been reported for two dehydrins from Arabidopsis^34^, while a LEA_4 protein from an anhydrobiotic nematode was not able to stabilize enzymes during heating^21,30^. Our data indicate that it will be well worth testing further LEA_4 proteins for potential chaperone activity and to characterize this interesting function further. It has been proposed^34^ that the chaperone function of dehydrins may be due to a “molecular shield” mechanism that would prevent aggregation, however, experimental evidence for such a mechanism is still lacking. Also, since LDH is a tetramer in its functional form, TdLEA3 may also be able to prevent dissociation of the monomers under heat stress or allow functional reassembly upon cooling. Further research will be necessary to resolve this question.

## Materials and Methods

### Plant material and stress treatment

Seeds of durum wheat (*Triticum turgidum* L. subsp. *durum*) cultivar OmRabia3 were provided by the Tunisian Agronomic Research Institute. After sterilization and germination, four days-old seedlings were transferred to containers with modified half-strength Hoagland’s solution^51^ to grow for 10 days under greenhouse conditions (25 ± 5 °C, 16 h photoperiod at 280 µmol photons m^−2^ s^−1^ and 60 ± 10% relative humidity). For stress treatments, seedlings were transferred to a solution containing 200 mM NaCl. After three days of stress application, plants were immersed in liquid nitrogen and then stored at −80 °C for RNA isolation.

### Isolation, expression and purification of durum wheat TdLEA3 protein

Total RNA was isolated from the durum wheat seedlings using Trizol Reagent (TaKaRa, China). cDNA was synthesized by M-MLV reverse transcriptase (Invitrogen) at 37 °C for 1 h using oligo-dT_18_ primers. The *TdLEA3* cDNA was subsequently amplified using PFU polymerase (Fermentas) and primers designed on the basis of the gene sequence of *Triticum aestivum* LEA3 (AY148492): 5′-ATGGCCTCCAACCAGAACCA-3′ and 5′-CTAGTGATTCCTGGTGGTGGT-3′. Amplified products were purified and cloned into the pGEM-T Easy vector system (Promega) and successful isolation of *TdLEA3* DNA was confirmed by restriction analysis and sequencing. The 5′ and 3′ regions of *TdLEA3* cDNA were verified with the 5′- and 3′-RACE technique using the ‘First choice RLM Race kit’ according to the instructions of the manufacturer (Ambion). The following primers were used (5′ to 3′): R5-TdLEA3: ATCACCTGCCCGGTCTTCT and R3-TdLEA3: ACAACGCCACCAAGGACAC. The full-length open reading frame of *TdLEA3* (GenBank accession: KR698795) was amplified from the durum wheat variety OmRabia3 cDNA using the following primers corresponding to the 5′ and 3′ ends and containing *EcoR*I restriction sites at their ends: TdLEA3: 5′-GAATTCATGGCCTCCAACCAGAAC-3′ and TdLEA3: 5′-GAATTCGTGATTCCTGGTGGTGGT-3′.The *TdLEA3* ORF was cloned into the *EcoR*I site of the *Escherichia coli* expression vector Pet28a. Recombinant protein production of TdLEA3 in *E*. *coli* BL21 (DE3) was induced by 1 mM IPTG at 37 °C, cells were grown for 4 h, lysed in NENT buffer (100 mM NaCl, 1 mM EDTA, 0.5% NP40, 20 mM Tris-HCl, pH 7.9) containing the protease inhibitor phenylmethylsulfonyl fluoride and purified by HisLink Protein Purification Resin according to the manufacturer’s (Promega) instructions. The concentration of the recombinant protein was determined spectrophotometrically at 280 nm using a molar extinction coefficient calculated from the amino acid sequence using an online tool (http://encorbio.com/protocols/Prot-MW-Abs.htm).

### *In-silico* analysis

Molecular mass, theoretical pI and grand average of hydropathy (GRAVY) of TdLEA3 were assessed by the ExPASy-ProtParam tool (http://web.expasy.org/protparam/). Sequence identities were determined using BLAST on the NCBI web server (http://blast.ncbi.nlm.nih.gov/Blast.cgi). The Kyte–Doolittle scale^48^ was used for hydrophobicity plots. Multiple sequence alignment was performed using Clustal Omega with default parameters. The tendency of disorder of the investigated protein was predicted by IUPRED (http://iupred.enzim.hu/). Secondary structure was predicted using the SOPMA tool (https://npsa-prabi.ibcp.fr/cgi-bin/npsa_automat.pl?page=/NPSA/npsa_sopma.html). Protein conserved motif analysis was conducted using RADAR (https://www.ebi.ac.uk/Tools/pfa/radar/). The resulting α-helical domain of TdLEA3 was visualized in a helical wheel projection using the HELIQUEST web tool (http://heliquest.ipmc.cnrs.fr/cgi-bin/ComputParams.py).

### SDS-PAGE and western blot analysis

Five µg of protein per sample were separated by SDS-PAGE^52^. Protein bands were either visualized by Coomassie staining or electroblotted onto a nitrocellulose membrane (Schleicher and Schuell) using a semi-dry blotting system (Bio-Rad). Nonspecific binding sites were blocked with PBST (Tween 20 diluted 1000-fold in 10x PBS + 5% non-fat dry milk powder). The blot was probed with peroxidase-coupled anti-His tag antibody (Abcam) in a 1:2000 dilution in PBST with 1% non-fat dry milk.

### CD spectroscopy

CD spectra were obtained with a Jasco-815 spectropolarimeter (Jasco Instruments). Protein solutions containing approximately 0.5 mg/ml protein in H_2_O were measured in a 0.1 mm path-length cuvette. Protein concentrations were estimated from the absorbance at 193 nm as described previously^53^. For the measurement of dry samples, 20 µl of 2 mg/ml protein dissolved in H_2_O were spread on CaF_2_ windows and dried in a desiccator over silica gel over night at 28 °C. We have previously verified the accuracy of our secondary structure determination of proteins in the dry state using reference proteins of known structure^15^. Measurements and data analysis were performed as described in detail previously^15^.

### FTIR spectroscopy

FTIR measurements were performed on a Perkin Elmer GX2000 FTIR spectrometer equipped with a vacuum cuvette in the IR beam (Specac, Worthington, UK, ^54^). Samples (2 mg protein/ml) dissolved in D_2_O were analyzed between two CaF_2_ windows separated by a 0.01 mm Teflon spacer at ambient pressure^55^. Dry samples were prepared by spreading 50 µl of protein solution in H_2_O on a CaF_2_ window and drying them as described above for CD spectroscopy. These samples were measured under vacuum as described before^54,56^. To measure FTIR spectra of TdLEA3 at different relative humidities, dry protein samples on CaF_2_ windows prepared as described above were incubated for 24 h over different saturated salt solutions or over D_2_O. Samples were then covered by a second CaF_2_ window and measured under the same conditions as the fully hydrated samples^16^. Three samples per condition were measured and 32 spectra were coadded per sample. Changes in the secondary structure of the protein were estimated by analyzing the Amide I peak (1700–1600 cm^−1^) of the FTIR spectra using Spectrum 10.4.3 software (PerkinElmer, Rodgau, Germany). Spectra from the three replicates were averaged and smoothed in Kaleidagraph 4.5.0 (Synergy Software, Reading, PA, USA) using a 10 point sliding window.

### LDH protection assay

Lactate dehydrogenase (LDH) from bovine heart was obtained from Sigma–Aldrich and diluted in 10 mM sodium phosphate, pH 7.4 following the manufacturer’s recommendations. One µl of 20 µg/µl LDH was added to a total of 20 µl of buffer with or without TdLEA3 or BSA at a mass ratio of 1:1, 1:10, 1:20 and 1:40 (LDH: LEA/BSA). Samples were vacuum-dried in a Speed Vac (Savant Speed Vac Plus Model SC110A Concentrator) to a final volume of 6 µl and rehydrated by the addition of 14 µl of buffer. To test the effect of freezing on LDH activity, samples were frozen in liquid nitrogen for 1.5 min and thawed at room temperature. Freeze-thaw cycles were performed up to four times. In heating assays, samples were treated at 48 °C for up to 30 min. To determine enzyme activity, 1 ml of freshly prepared assay buffer (10 mM sodium phosphate (pH 7.4), 2 mM NADH, and 10 mM pyruvic acid) was added to the LDH samples. NADH oxidation was monitored by measuring the absorbance at 340 nm over 3 min, during which the reaction rate was linear. The rate of absorbance decrease was then used to calculate activity (∆OD/min) × 8095 = U/l (Biomaghreb kit). All samples were assayed in triplicate.

### *In vitro* protein aggregation assay

LDH was chosen as an example of an aggregation-prone protein during desiccation–rehydration, freezing-thawing and upon heating. Aggregation of LDH was monitored in a spectrophotometer (JENWAY, 7305) as the change in absorbance at 340 nm. Drying was performed as described above with the following modifications. One hundred µl of LDH (1 mg/ml) were partially dried in the absence or presence of TdLEA3 or BSA at 1:1 and 1:2 (enzyme: protein mass ratio) for 2.5 h until reaching 30% of the initial sample volume. Subsequently, samples were rehydrated to the original volume with water. In heating assays, samples were treated at 80 °C for 20 min. To test the effect of freezing on LDH activity, samples were frozen in liquid nitrogen for 1.5 min and thawed at room temperature. Freeze-thaw cycles were performed up to three times. All samples were assayed in triplicates.

## Data Availability

The datasets generated and analysed during the current study are available from the corresponding author on reasonable request.
